# Problem-solving versus cognitive restructuring of medically ill seniors with depression (PROMISE-D trial): study protocol and design

**DOI:** 10.1186/1471-244X-12-207

**Published:** 2012-11-22

**Authors:** Louise Sharpe, Catherine B Gittins, Helen M Correia, Tanya Meade, Michael K Nicholas, Patrick J Raue, Sarah McDonald, Patricia A Areán

**Affiliations:** 1 School of Psychology, University of Sydney, Sydney, Australia; 2 School of Psychology, Murdoch University, Perth, Australia; 3 School of Psychology, University of Western Sydney, Sydney, Australia; 4 School of Medicine, University of Sydney, Sydney, Australia; 5 Pain Management Research Institute, University of Sydney at Royal North Shore Hospital, Sydney, Australia; 6 Weill Medical College, Cornell University, New York, USA; 7 Department of Psychiatry, University of California, San Francisco, USA

**Keywords:** Depression, Older adults, Chronic illness, Cognitive-behavioural therapy, Problem-solving therapy, Psychotherapy

## Abstract

**Background:**

With an ageing population in most Western countries, people are living longer but often with one or more chronic physical health problems. Older people in physically poor health are at greater risk of developing clinical depression. Cognitive Behavioural Therapy (CBT) and Problem Solving Therapy (PST) have both been found to be efficacious in treating late-life depression, however patients with “multi-morbidity” (i.e. more than one chronic condition) are often excluded from these trials. The aim of this study is to compare the efficacy of CBT and PST in treating older adults who have one or more chronic physical health conditions and a diagnosable depressive disorder. This study will be the first to explicitly target the treatment of depression in older people in primary care settings presenting with a range of health problems using behavioural interventions.

**Methods/design:**

The PROMISE-D study is a randomised controlled trial of two evidence-based treatments for late-life major or minor depression for patients who also have at least one co-morbid chronic health problem. Participants will be randomised to two active interventions (PST or CBT) or enhanced treatment-as-usual (E-TAU). Primary outcomes will be depression diagnostic status and severity of depression (according to the Hamilton Depression Rating Scale and the Geriatric Depression Scale). Secondary outcomes will be anxiety severity, quality of life and health care utilisation. Assessments will be conducted by a researcher who remains blind to the patient’s treatment allocation and will be conducted pre and post-treatment and at six and 12 months follow-up. Health care utilisation will be assessed throughout a two year period following entry to the trial. Executive function, rumination and emotion regulation will also be measured to determine the impact of these factors on treatment response in two treatment groups.

**Discussion:**

Multi-morbidity, the experience of two or more chronic health problems, is becoming an increasing problem internationally, particularly amongst the elderly. Evidence-based psychological treatments exist for late-life depression and these have been shown to be effective for participants with individual health problems and depression. However, there are no studies that have compared the two leading psychotherapies shown to be effective in the treatment of late-life depression. In addition, many trials of psychotherapy with older adults exclude those with multi-morbidity. Hence, this trial will confirm whether CBT and PST are efficacious in the treatment of depression in the context of complex medical needs and determine which of these two interventions is most efficacious.

**Trial registration:**

ACTRN12612000854831

## Background

Older age is associated with an increased risk of depression [1]. A systematic review revealed six consistent factors associated with depression in older people. Excepting female gender, physical health and functional impairment were the strongest predictors of late-life depression [2]. Increased rates of depression have been found in older patients with a range of illnesses [3-6]. Indeed, one study demonstrated 83% of those over 75 had more than one health problem, and a psychological and health problem was the second most common co-morbidity [7]. Of even more concern, research confirms that physical illness also increases the risk of suicide amongst depressed older adults [8].

Despite a large number of trials of psychological therapies in young depressed patients [9], there are far fewer studies in older people. The number of trials in the literature has been increasing, which has given rise to numerous reviews of the efficacy of psychotherapeutic interventions for late-life depression [10-14]. Although those reviews varied in which interventions were found to be definitely efficacious, there is some general consensus.

Cognitive Behavioural Therapy (CBT), a therapy based on changing unhelpful beliefs and increasing activity as a treatment for depression, is the most well researched therapy in the treatment of late-life depression [12]. In their Cochrane Review, Wilson and colleagues [12] concluded that CBT was the only definitely efficacious therapy for late-life depression. They used very stringent criteria for trial inclusion, but nonetheless, on this point there is considerable consistency between reviews. That is, CBT is a proven effective treatment for late-life depression [10-14].

In the Cochrane review, Problem Solving Therapy (PST) was considered under the umbrella of CBT [10,12]. However, there are important differences. CBT focuses on restructuring and challenging beliefs that are associated with low mood and behavioural activation, whereas PST focuses on dealing with the problems associated with daily life and shifting negative cognitions into goal directed activity [15]. One review of psychological treatments in older adults [14] that distinguished between PST and CBT, concluded that PST was probably efficacious. However, since that review, there have been a number of additional, well controlled trials that have evaluated PST as a treatment for late-life depression, each of which has supported the efficacy of PST [16-21]. Hence, PST can be regarded as meeting the criteria for a definitely efficacious treatment [22]. Furthermore, in a meta-analysis comparing psychotherapies for adult depression, PST was found to have the lowest drop out rates of any of the therapies, making it a highly acceptable intervention among people suffering from depression [23].

Although there is good evidence to support the efficacy of CBT and PST for the treatment of later life depression, there is a need for further research [13]. Which of these treatments is most efficacious remains unclear. To date, no trials have compared CBT with PST [10]. Research comparing those treatments shown to be most efficacious for the treatment of late life depression is needed in order to ensure that patients are offered the most efficacious intervention. Large trials comparing these treatments can also investigate whether different patients benefit from different treatments. If the different treatments are suited to patients with different characteristics, there may be ways in which interventions can be matched to those patients most likely to benefit from them.

Similarly, there is a pressing need for outcome studies for depressed patients with complex needs. In their meta-analysis, Pinquart and colleagues [10] found that effect sizes were smaller in studies that included people with a co-morbid medical illness. However, there are few trials of the treatment of late-life depression for people with a range of co-morbid physical health problems and patients with multi-morbidity (i.e. two or more concurrent physical and/or psychological disorders are often excluded from the published trials). Only the PROSPECT [24] and IMPACT [18] trials have studied treatment of depression in older medical patients, however, the interventions studied were not exclusively psychological. In these studies, patients were offered a choice of either medication or behavioral treatment. There are, however, some studies in patients with specific illnesses that show that CBT is effective in reducing depressive symptomatology in patients with chronic illnesses often seen in older adults. For example, CBT is successful in reducing depressive symptoms in arthritis and preventing the development of new “cases” of depression over 18 months [25,26]. Similarly, recent research shows that CBT for Chronic Obstructive Pulmonary Disease (COPD) is successful in improving depressive symptoms [27].

Despite these encouraging results, neither study specifically included patients who met criteria for major or minor depression. Indeed, there appear to be no studies of CBT in late life depression where patients were included on the basis of a diagnosis of clinical depression and a comorbid health problem. Two studies investigated the efficacy of CBT for patients scoring above a cut-off score on questionnaires. Both studies (a small trial in stroke survivors [28] and a larger trial in Chronic Obstructive Pulmonary Disease patients [29]) failed to find a benefit of CBT. However, in the stroke study, patients were recruited on the basis of their scores on a screening test (the Beck Depression Inventory), known to be inflated in physically ill populations. Of those allocated to CBT, 69% met criteria for a depressive disorder, in contrast to only 43% of the control group. These initial differences could have masked any treatment effects. There are also problems with the Chronic Obstructive Pulmonary Disease study. Kunik et al. [29] included patients with heightened levels of depression OR anxiety and attempted in an 8 x 1 h program to address both symptoms in groups of 10 patients. The cognitive component (i.e. cognitive restructuring) involved only two sessions, and this may not be sufficient, particularly in older adults, to have conferred benefit [10]. While these studies have some problems, the null findings highlight the importance of trials to confirm that the efficacy of CBT extends to those with comorbid medical problems and late-life depression.

There is more research about the efficacy of PST in the treatment of late life depression in physically ill patients. One early trial in older adults with medical illness found positive results [30], but was uncontrolled. There have since been a number of large trials in the context of particular health conditions. Areán and her colleagues have shown that PST can improve depression in patients with arthritis [31] and in older disabled people with cognitive dysfunction [16,32]. This provides good evidence that at least in some disorders, PST can ameliorate depressive illness even where complicated by the presence of one serious, chronic health problem. Additionally, PST has been found to be effective in disabled older adults (many of whom have chronic illnesses) [33,34] and reduces the risk of post stroke depression [35], as well as reducing depression symptoms in cancer patients [36]. However, the question of whether PST can be applied across the range of health problems common in later life, particularly in those with multi-morbidity, and whether it is as effective as CBT, remains unclear.

### Aims and hypotheses

Although there have been numerous trials of psychological interventions for patients with late-life depression in the past decade, there continue to be major gaps in the literature. Firstly, few studies aim to treat depression in the context of ill health, despite the fact that many older adults have both depression and one or more chronic physical illnesses. There are also few studies that compare different psychotherapies for late-life depression. The PROMISE-D trial aims to bridge these gaps. Specifically, the aim of the study is to evaluate two psychological therapies (PST and CBT), both of which are definitely efficacious for the treatment of late-life depression, for patients with one or more co-morbid physical illness.

We hypothesise that PST and CBT will be superior to a wait-list control group in the treatment of depression for patients with co-morbid health problems. We also aim to determine what baseline characteristics might best predict outcome in each treatment condition. Based on the hypothesised mechanisms of PST, we expect that patients with executive dysfunction (poor score on the Stroop Interference Task [37]) will respond better to PST than CBT [16]. In contrast, we expect patients with excessive rumination (measured by the Ruminative Response Scale; RRS [38]) and patients with affective dysregulation (measured by the Emotion Regulation Questionnaire; ERQ [39]) to respond better to CBT than PST [39,40].

## Methods/design

**Figure 1:**
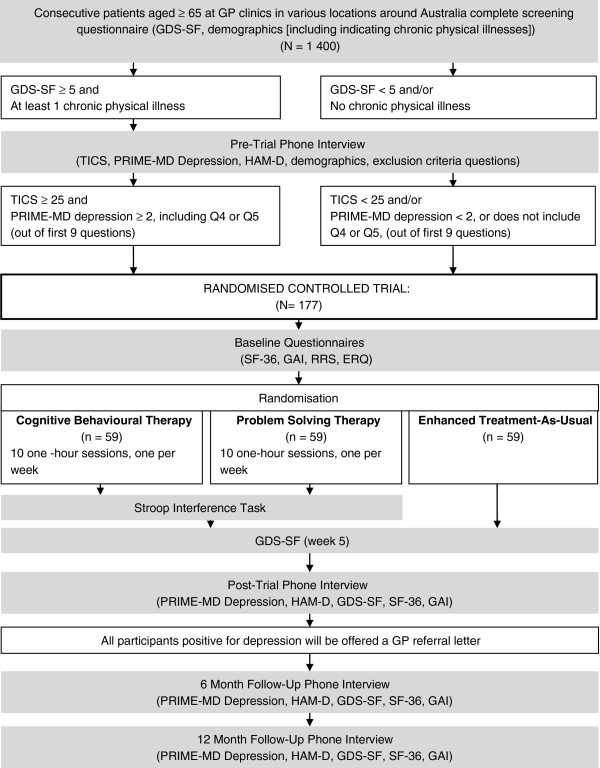
Outline of study design.

The procedure of the PROMISE-D trial is illustrated in Figure 1. This study has been designed in accordance with CONSORT criteria [41] and is a randomised controlled trial (RCT) registered with the Australian New Zealand Clinical Trial Registry. Ethical approval has been granted for this study by the University of Sydney Human Research Ethics Committee.

### Participants

Consecutive adults aged 65 years and older presenting at GP surgeries will be screened for the study. The inclusion criteria are (1) one or more chronic physical illness; (2) a diagnosis of major or minor depressive disorder or dysthymia; (3) if taking anti-depressant medication, a stable dose for > 8 weeks; (4) have not received electroconvulsive therapy (ECT) within 6 months (to exclude memory problems) and (5) sufficient English to complete questionnaires and take part in therapy. Participants will be excluded if they have (1) suicidal intent requiring emergency care; (2) alcohol or drug abuse or dependence; (3) a history of bipolar disorder or cyclothymia; (3) a psychotic illness; (4) cognitive deficits (< 25 on the Telephone Interview for Cognitive Status [TICS] [42]); or (5) have received psychotherapy within the last six months.

Participants will be recruited at their GP surgery when they attend for an appointment. Based on the prevalence of physical health problems amongst older people (60%) (and depressive symptoms high enough to warrant screening amongst those [30%]), and the likely rate of actual disorder from those who screen positive (70%), we anticipate that approximately 1400 older people will need to be screened in order to achieve the 177 participants required for the RCT.

### Procedure

GP surgeries will be sent a letter inviting them to participate in the study. If they agree to be involved, consecutive attendees at participating surgeries who are 65 and over will be asked to complete a one-page double-sided screening questionnaire and post it in a locked box located in the surgery. The screening questionnaire will consist of the Geriatric Depression Scale- Short Form (GDS-SF [43]) and some basic demographic questions (age, gender). Respondents will also be asked to indicate, from a list provided, which chronic illnesses (if any) they have. People who prefer not to complete the questionnaire will be asked to put the unanswered page into the locked box to provide an estimate of how many individuals refused to take part. Participants will be informed that if they give the researchers their details and are eligible for the study, that the researchers will contact them directly.

Those participants who score ≥ 5 on the GDS-SF [43] and have at least one chronic illness will be telephoned by the researchers and asked if they would like to participate in a phone interview. The interview will be used to determine whether they meet the criteria for major or minor depression using the Primary Care Evaluation of Mental Disorders - Depression subscale (PRIME-MD [44]). Their cognitive status will also be assessed via the Telephone Interview for Cognitive Status [42] and more detailed demographic questions will be asked. If they meet criteria for a clinical depression (major or minor) as assessed by the PRIME-MD, the Hamilton Rating Scale for Depression (HAM-D [45]) will also be conducted and participants will be offered the opportunity to take part in the RCT.

Participants who complete the phone interview and are eligible for the RCT will be told about the trial at the end of the phone interview. Those individuals interested in participating will be sent a participant information statement, consent form and the baseline questionnaires (Short Form (36) Health Survey; SF-36 [46]; Geriatric Anxiety Inventory [47]; Ruminative Response Scale [38]; Emotion Regulation Questionnaire [39]). Upon receipt of their consent form and baseline questionnaires, participants will be randomised, by an independent researcher, into one of three conditions: CBT, PST or E-TAU (described below). Medical files will be accessed to get details of current medications (including anti-depressant medication) and to confirm their diagnoses. Participants will then be contacted by a member of the research team and informed of their condition. If allocated to CBT or PST, the researcher will arrange an appointment for their first therapy session, which will be conducted at a local health clinic. During the first session participants in the PST and CBT conditions will complete the Stroop Interference Task [37].

Treatment consists of 10 one hour weekly sessions. All participants will have their depressive symptoms monitored via the administration of the GDS-SF midway through the trial period (i.e. after 5 weeks). If any individual demonstrates a reliable deterioration [48], from trial entry, in GDS-SF score they will be contacted by the research team to determine their ability to continue the trial and to ensure their safety. Post-treatment assessment using PRIME-MD Depression module, GDS-SF and the HAM-D will be conducted. Participants will also be asked to complete the SF-36 to assess their quality of life and the Geriatric Anxiety Inventory to assess co-morbid anxiety symptoms at post-treatment. Follow-up assessments will be conducted six and 12 months after the trial. Data on health care utilisation will be gained from Medicare and the Pharmaceutical Benefits Scheme in order to determine the cost efficacy of the interventions in the two years following treatment.

### Randomisation

Randomisation will be stratified by diagnosis (major depressive episode or minor depression/dysthymia). This will be performed by an independent researcher who has no other involvement in the trial, using computer-generated random numbers.

### Therapist training

Clinicians will be registered psychologists, with training in cognitive-behavioural therapy. All therapists will complete a four-day workshop during which they will receive group training in the therapy techniques, with 2 days devoted to PST and 2 days devoted to CBT. The training programs for the two therapies will be as closely matched in structure as possible to allow for comparability across conditions, and recommendations for maintaining treatment fidelity in randomised trials [49] will be followed. PST training will be conducted by PJR and LS and CBT training will be conducted by LS and HMC.

Prior to the training week, all therapists will receive a manual for PST and a manual for CBT which will outline the protocols for treatment. Both PST and CBT will be specifically targeted at treating older adults with chronic physical illnesses. At the start of the first PST session, therapists will be given an overview of PST. Likewise, during the first CBT session an outline of CBT will be provided. For each therapy type, eight role-play scenarios will be conducted in which the trainers act out the roles of patients. Trainees will act as therapists in around two role-plays each. Trainees will also listen to audio or video recordings of PST and CBT sessions and will then be required to analyse the strengths and weaknesses of the recorded sessions. A random selection of PST audio recordings will be evaluated by an independent researcher using the PST Adherence Form (PST-AS [50]), a session check list that determines the degree to which therapists apply the 7-PST steps in a therapy session to high quality. Similarly, the Cognitive Therapy Rating Scale [51] will be used to assess the quality of cognitive therapy administered. In addition, therapists in each condition will be asked to keep a self-reported fidelity checklist of all sessions. This will allow quality of sessions and average fidelity to be compared between the two treatment types.

### Trial conditions

Clinicians will follow strict manualised protocols under the supervision of LS.

#### Cognitive behavioural therapy

There are two primary components of CBT: behavioural activation and cognitive restructuring. Behavioural activation teaches patients about the relationship between activity and mood, such that lowering levels of activity results in reductions in mood, creating a vicious cycle often referred to as the lethargy circuit. Participants are encouraged to develop realistic goals that are associated with either mastery (i.e. the feeling of achievement for having completed a goal) or pleasure. Participants are also encouraged to monitor their moods in relation to their goals and other daily activities. Over the course of the therapy, participants are encouraged to gradually increase their involvement in activities. Cognitive restructuring helps patients learn to identify unhelpful thoughts and beliefs in daily situations. Particular strategies are learned to help challenge the validity of their assumptions and to develop more helpful and realistic appraisals in daily situations. Behavioural experiments are developed within session in order to test out unhelpful beliefs. These challenging strategies are applied in daily situations to help participants to gain better control over their thoughts and subsequently their mood.

#### Problem solving therapy

PST teaches patients structured problem solving strategies that they learn to apply to everyday difficulties, as well as to more significant adverse life events, such as divorce or serious medical illness. The program will specifically target those problems arising from their medical conditions. In the first session the patient will receive psychoeducation on depression in general and learn specifically about the mechanics of PST and why it is effective. Therapists will guide patients to generate a list of problems that they are currently experiencing in their lives and select and clearly define a moderately challenging one to focus on. Patients will then identify a goal, related to that problem, that they would like to achieve and brainstorm strategies to meet the goal, producing as many solutions as possible. Following this, patients assess the pros and cons of each solution they have created and choose one or more to implement. They then generate an action plan containing steps by which to carry out this solution. At subsequent sessions the action plan is reviewed and the success (or lack of success) at achieving the goal is discussed, and a new solution is developed if necessary.

#### Enhanced treatment-as-usual

Participants in the E-TAU condition will not receive any specified treatment as a result of the trial. However, they will be free to pursue treatment as usual during the course of the 10 week trial period. Any treatments initiated during the 10 week treatment phase of the trial will be carefully monitored. However, GPs will receive no information about the participant’s mood during the 10 week treatment phase of the trial. Because it would unethical to have assessed a person with a depressive disorder and provide no treatment for such a lengthy period of time, once post-treatment assessments are completed, GPs of participants who still meet criteria for a depressive disorder will be informed of the participant’s depression status.

### Outcome measures

The primary outcome will be diagnoses of depression according to the PRIME-MD Depression module. In order to allow for multi-modal assessment we will also measure participants’ depression using the GDS-SF [43] and clinician-rated severity according to the HAM-D [45]. Two secondary outcomes will be measured. Quality of life will be assessed with the SF-36 [46] and anxiety will be measured using the Geriatric Anxiety Inventory [47]. These assessments will be conducted by a researcher who remains blind to the participant’s treatment allocation. Blindness will be confirmed by having the independent researcher guess the group that they think each participant belongs to at the end of the treatment period.

As we are interested in which factors affect treatment success, we will also be taking a number of process measures. Executive function will measured using the Stroop Interference Task [37]. For logistical reasons only participants in PST and CBT conditions will be administered the Stroop task because it requires face-to-face administration. The Stroop will be used as a predictor of treatment outcome and not an outcome measures. All participants will be assessed for level of rumination (via the Ruminative Response Scale [38]) and affective regulation (via the Emotion Regulation Questionnaire [39]).

Additionally, demographic variables such as ethnicity and level of education will be recorded, as well as more specific information about previous depressive diagnoses and treatment. In order to confirm details of their chronic illness(es), RCT participants will also be asked to consent to the research team accessing their medical files.

### Analysis

#### Sample size

177 participants will be recruited to the RCT. In order to achieve 95% power and α = 0.05, 47 participants per group are needed. Allowing for 20% attrition, this requires 59 participants per group.

#### Statistical analysis

There are 4 measurement times, of which 3 are post-intervention. Linear mixed models will be used to account for the hierarchical, non-independent nature of the data: repeated measures on patients nested within clusters (primary care services). Baseline outcome measures (e.g., HAM-D and GDS-SF) will be used as a covariate, which improves efficiency [52]. These models will allow for 1) comparing patterns of change over time by testing the intervention group by time interaction and 2) estimating and testing differences in outcome measures between groups at time points of interest via linear contrasts. Reporting will follow the CONSORT statement [41].

##### Sample size

The primary outcomes are a diagnosis of depression according to PRIME-MD and severity of depression according to the HDS and GDS. In a recent meta-analysis, the average effect size for CBT (including PST trials) was 1.06 and for PST was 1.00 [10]. To be conservative, we have used the smaller ES of 1.00. According to G-power, we would need only 25 participants per group have 95% power to detect a significant difference (p < 0.05) between the 2 active treatments and the control condition [53]. However, we are also interested in the relative efficacy of each of the 2 interventions. Given that these two treatments have never been compared in any clinical setting, we determined that to be conservative, we should power the study to identify even small differences in effect sizes (Cohen’s D = 0.25) between the two treatments. In order to be able to observe a difference between the two groups of 0.25 with 80% power and a significance level of 0.05, we need 59 participants per group. That is, 177 participants in total.

## Discussion

In Australia, as in the rest of the developed world, we have a large, ageing population. According to the Australian Bureau of Statistics [54], the number of people older than 65 in Australia increased by 170% over the past 2 decades, compared to only a 31% increase in the rest of the population. Research shows that as the ageing population increases, the incidence of both chronic medical illness and late life depression is also increasing. Moreover, the presence of depression amongst the physically ill is known to complicate the physical illness itself. Further, older, depressed people with physical illnesses fare more poorly than those without co-morbid physical problems in therapy. To date, no psychological treatment has been shown to be definitely efficacious with this group of patients with multi-morbidity, and who are amongst the most vulnerable in our community.

The proposed study will be the first to compare two evidence-based interventions in the treatment of late-life depression in older adults who also have one or more chronic medical conditions. This novel and timely research will implement a methodologically rigorous RCT of two interventions which already have substantial evidence of efficacy in treatment of late-life depression in healthy people. The eventual goal of this study is to distribute an evidence-based, manualised, psychological intervention for older people with chronic health problems in primary care.

### Strengths of the study

The team who have developed the PROMISE-D trial include senior, experienced researchers who have previously developed manuals for PST in the treatment of late-life depression (PAA and PJR) and cognitive behavioural treatments for people with health problems (LS and MKN) and tested these in randomised controlled trials. These researchers have extensive experience in training psychologists in these strategies and hence the PROMISE-D trial will provide a valid test of these interventions.

The enhanced treatment-as-usual arm will ensure that the pre to post treatment changes are able to determine the efficacy of these interventions in comparison to treatment-as-usual in the short-term (as no correspondence will occur between the GPs and researchers). However, the fact that GPs will be given the opportunity to respond to an identified case of depression following the treatment phase of the PROMISE-D trial, means that this trial will provide a stringent test of the long-term efficacy of these two interventions. Further, this should help us ascertain whether, in the longer term, these interventions are more efficacious than routinely offered treatments if depression were identified in practice.

### Challenges

We envisage that the greatest challenge in this trial will be recruiting the sample. There are a number of potential barriers to recruitment in this study. Firstly, recruiting busy GP practices to take part in this study may prove challenging. However, we have designed the PROMISE-D trial to require minimal input from surgeries, such that they need only hand out a single form to all people over 65. Involvement in the trial will result in considerable benefits to older patients with multi-morbidity, a group that is particularly difficult to treat. The benefits include screening for depression and treatment of depression (in 66% of cases) without cost to the patient or GP. We have also agreed to offer free training to the psychologists attached to any GP surgeries at the end of the trial in whichever intervention proves most efficacious. This will also ensure long-term implementation of effective psychological care for these patients.

There are also barriers to recruitment of this particular patient group. Older people with chronic physical illnesses and depression may be difficult to recruit because motivation may be problematic, as related to their mood difficulties. Furthermore, mobility issues may also compromise their ability to attend sessions. To deal with these issues we will attempt to see patients in their GP surgery to minimise travel and have some funding to compensate participants for direct travel costs, where necessary.

### Conclusion

In summary, although recent evidence shows that both Cognitive Behavioural Therapy and Problem Solving Therapy are effective in the treatment of late-life depression, many of trials exclude patients with complex medical needs. However, multi-morbidity (the co-morbidity of two or more chronic illnesses) is increasingly common in Australia and in most Western countries. Whether these psychological treatments work for older patients with one or more chronic physical health problems and a diagnosis of depression is currently unknown. Further, whether CBT or PST is more effective has not been tested in the late life depression literature. Information about which of these treatments is most effective in this group of older patients with multi-morbidity will inform the provision of evidence-based care for this particularly vulnerable group of patients.

## Abbreviations

PROMISE-D: Problem-solving versus cognitive Restructuring Of Medically Ill SEniors with Depression; CBT: Cognitive Behavioural Therapy; PST: Problem Solving Therapy; E-TAU: Enhanced-Treatment-As-Usual; RCT: Randomised Controlled Trial; TICS: Telephone Interview for Cognitive Status; GDS-SF: Geriatric Depression Scale-Short Form; PRIME-MD: Primary Care Evaluation of Mental Disorders; HAM-D: Hamilton Rating Scale for Depression; SF-36: Short Form (36) Health Survey.

## Competing interests

The authors declare no competing interests.

## Authors’ contributions

LS, PAA, MKN, TM and HMC were investigators on the successful grant application. This paper was drafted by LS and CBG, which was then modified by all other authors. All authors contributed to the design of the study protocol. The PST intervention was developed by PAA and PJR and the CBT intervention was developed by LS. The final manuscript was read and approved by all authors.

## Pre-publication history

The pre-publication history for this paper can be accessed here:

http://www.biomedcentral.com/1471-244X/12/207/prepub
